# 2-Hy­droxy-4-meth­oxy­benzaldehyde thio­semicarbazone

**DOI:** 10.1107/S1600536810029594

**Published:** 2010-08-04

**Authors:** Yu-Mei Hao

**Affiliations:** a Department of Chemistry, Baicheng Normal University, Baicheng 137000, People’s Republic of China

## Abstract

The title Schiff base compound, C_9_H_11_N_3_O_2_S, was prepared by the reaction of equimolar quanti­ties of 2-hy­droxy-4-meth­oxy­benzaldehyde with thio­semicarbazide in methanol. The mol­ecule adopts a *trans* configuration with respect to the azo­methine group and an intra­molecular O—H⋯N hydrogen bond generates an *S*(6) ring. In the crystal structure, mol­ecules are linked through inter­molecular N—H⋯O and N—H⋯S hydrogen bonds, forming a three-dimensional network.

## Related literature

For a related structure and background references, see: Hao (2010 ▶). For reference structural data, see: Allen *et al.* (1987 ▶).
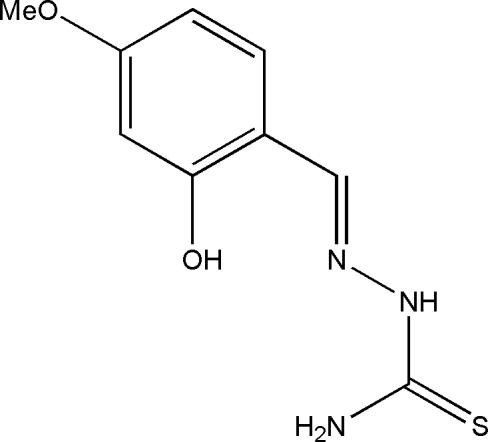


## Experimental

### 

#### Crystal data

C_9_H_11_N_3_O_2_S
*M* *_r_* = 225.27
Monoclinic, 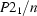

*a* = 4.929 (1) Å
*b* = 10.519 (2) Å
*c* = 20.357 (3) Å
β = 92.838 (2)°
*V* = 1054.2 (3) Å^3^
*Z* = 4
Mo *K*α radiation
μ = 0.29 mm^−1^
*T* = 298 K
0.17 × 0.13 × 0.12 mm

#### Data collection

Bruker SMART CCD diffractometer
Absorption correction: multi-scan (*SADABS*; Sheldrick, 1996 ▶) *T*_min_ = 0.952, *T*_max_ = 0.966
5879 measured reflections
2247 independent reflections
1650 reflections with *I* > 2σ(*I*)
*R*_int_ = 0.030

#### Refinement

*R*[*F* ^2^ > 2σ(*F* ^2^)] = 0.040
*wR*(*F* ^2^) = 0.101
*S* = 1.04
2247 reflections
147 parameters
4 restraints
H atoms treated by a mixture of independent and constrained refinement
Δρ_max_ = 0.18 e Å^−3^
Δρ_min_ = −0.23 e Å^−3^

### 

Data collection: *SMART* (Bruker, 2002 ▶); cell refinement: *SAINT* (Bruker, 2002 ▶); data reduction: *SAINT*; program(s) used to solve structure: *SHELXS97* (Sheldrick, 2008 ▶); program(s) used to refine structure: *SHELXL97* (Sheldrick, 2008 ▶); molecular graphics: *SHELXTL* (Sheldrick, 2008 ▶); software used to prepare material for publication: *SHELXL97*.

## Supplementary Material

Crystal structure: contains datablocks global, I. DOI: 10.1107/S1600536810029594/hb5575sup1.cif

Structure factors: contains datablocks I. DOI: 10.1107/S1600536810029594/hb5575Isup2.hkl

Additional supplementary materials:  crystallographic information; 3D view; checkCIF report

## Figures and Tables

**Table 1:** Hydrogen-bond geometry (Å, °)

*D*—H⋯*A*	*D*—H	H⋯*A*	*D*⋯*A*	*D*—H⋯*A*
N3—H3*B*⋯O2^i^	0.89 (1)	2.26 (2)	2.998 (3)	141 (2)
N3—H3*A*⋯O1^ii^	0.88 (1)	2.23 (1)	3.076 (3)	162 (2)
N2—H2⋯S1^iii^	0.90 (1)	2.48 (1)	3.366 (3)	168 (2)
O1—H1⋯N1	0.82	1.99	2.700 (2)	145

Symmetry codes: (i) 
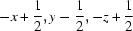
; (ii) 
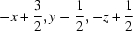
; (iii) 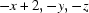.

**Fig. 1.**
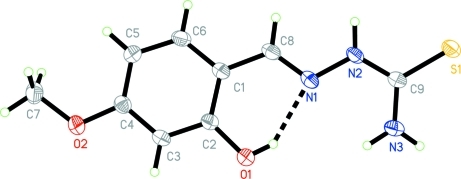
The molecular structure of the title compound with 30% probability ellipsoids. Intramolecular hydrogen bond is drawn as a dashed line.

**Fig. 2.**
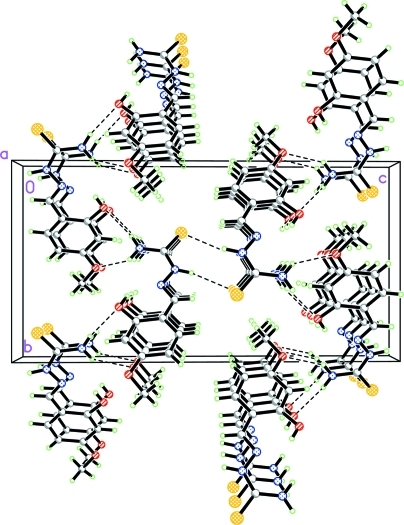
Molecular packing of the title compound with hydrogen bonds drawn as dashed lines.

## supplementary crystallographic information

### Comment

As a continuation of our structural studies of Schiff bases (Hao, 2010), in this paper, the title new Schiff base compound, (I), Fig. 1, is reported.

The molecule of the title compound adopts a *trans* configuration with respect to the azomethine group. All the bond lengths are within normal values (Allen *et al.*, 1987). There is an intramolecular O—H···N hydrogen bond (Table 1) in the molecule. In the crystal structure, molecules are linked through intermolecular N—H···O and N—H···S hydrogen bonds (Table 1), forming a 3D network (Fig. 2).

### Experimental

2-Hydroxy-4-methoxybenzaldehyde (0.1 mmol, 15.2 mg) and thiosemicarbazide (0.1 mmol, 9.1 mg) were refluxed in a 30 ml methanol solution for 30 min to give a clear colorless solution. Colorless blocks of (I) were formed by slow evaporation of the solvent over several days at room temperature.

### Refinement

H2, H3A and H3B were located from a difference Fourier map and refined isotropically, with the N—H and H···H distances restrained to 0.90 (1) Å and 1.53 (2) Å, respectively, and with *U*_iso_ restrained to 0.08Å^2^. Other H atoms were constrained to ideal geometries, with d(C—H) = 0.93-0.96Å, d(O—H) = 0.82Å, and with *U*_iso_(H) = 1.2*U*_eq_(C) and 1.5*U*_eq_(O1 and C7).

### Crystal data

**Table:**

C_9_H_11_N_3_O_2_S	*F*(000) = 472
*M**_r_* = 225.27	*D*_x_ = 1.420 Mg m^−^^3^
Monoclinic, *P*2_1_/*n*	Mo *K*α radiation, λ = 0.71073 Å
Hall symbol: -P 2yn	Cell parameters from 1566 reflections
*a* = 4.929 (1) Å	θ = 2.8–26.2°
*b* = 10.519 (2) Å	µ = 0.29 mm^−^^1^
*c* = 20.357 (3) Å	*T* = 298 K
β = 92.838 (2)°	Block, colorless
*V* = 1054.2 (3) Å^3^	0.17 × 0.13 × 0.12 mm
*Z* = 4	

### Data collection

**Table:**

Bruker SMART CCD diffractometer	2247 independent reflections
Radiation source: fine-focus sealed tube	1650 reflections with *I* > 2σ(*I*)
graphite	*R*_int_ = 0.030
ω scans	θ_max_ = 26.9°, θ_min_ = 2.0°
Absorption correction: multi-scan (*SADABS*; Sheldrick, 1996)	*h* = −6→6
*T*_min_ = 0.952, *T*_max_ = 0.966	*k* = −13→11
5879 measured reflections	*l* = −20→25

### Refinement

**Table:**

Refinement on *F*^2^	Primary atom site location: structure-invariant direct methods
Least-squares matrix: full	Secondary atom site location: difference Fourier map
*R*[*F*^2^ > 2σ(*F*^2^)] = 0.040	Hydrogen site location: inferred from neighbouring sites
*wR*(*F*^2^) = 0.101	H atoms treated by a mixture of independent and constrained refinement
*S* = 1.04	*w* = 1/[σ^2^(*F*_o_^2^) + (0.0453*P*)^2^ + 0.1092*P*] where *P* = (*F*_o_^2^ + 2*F*_c_^2^)/3
2247 reflections	(Δ/σ)_max_ < 0.001
147 parameters	Δρ_max_ = 0.18 e Å^−^^3^
4 restraints	Δρ_min_ = −0.23 e Å^−^^3^

### Special details

**Table:**

Geometry. All esds (except the esd in the dihedral angle between two l.s. planes) are estimated using the full covariance matrix. The cell esds are taken into account individually in the estimation of esds in distances, angles and torsion angles; correlations between esds in cell parameters are only used when they are defined by crystal symmetry. An approximate (isotropic) treatment of cell esds is used for estimating esds involving l.s. planes.
Refinement. Refinement of F^2^ against ALL reflections. The weighted R-factor wR and goodness of fit S are based on F^2^, conventional R-factors R are based on F, with F set to zero for negative F^2^. The threshold expression of F^2^ > 2sigma(F^2^) is used only for calculating R-factors(gt) etc. and is not relevant to the choice of reflections for refinement. R-factors based on F^2^ are statistically about twice as large as those based on F, and R- factors based on ALL data will be even larger.

### Fractional atomic coordinates and isotropic or equivalent isotropic displacement parameters (Å^2^)

**Table:**

	*x*	*y*	*z*	*U*_iso_*/*U*_eq_	
N1	0.6185 (3)	0.11719 (14)	0.11874 (8)	0.0391 (4)	
N2	0.7882 (3)	0.04027 (15)	0.08411 (7)	0.0420 (4)	
N3	0.9140 (4)	−0.06702 (18)	0.17762 (8)	0.0528 (5)	
O1	0.4001 (3)	0.22381 (13)	0.22458 (6)	0.0520 (4)	
H1	0.4909	0.1714	0.2055	0.078*	
O2	−0.2624 (3)	0.53431 (13)	0.19151 (6)	0.0483 (4)	
S1	1.12828 (11)	−0.14847 (5)	0.06920 (2)	0.0530 (2)	
C1	0.2729 (4)	0.27756 (17)	0.11180 (9)	0.0370 (4)	
C2	0.2464 (4)	0.29172 (18)	0.17967 (9)	0.0371 (4)	
C3	0.0662 (4)	0.37755 (18)	0.20376 (9)	0.0419 (5)	
H3	0.0521	0.3855	0.2490	0.050*	
C4	−0.0943 (4)	0.45216 (17)	0.16165 (9)	0.0381 (4)	
C5	−0.0757 (4)	0.43980 (18)	0.09409 (9)	0.0425 (5)	
H5	−0.1835	0.4891	0.0652	0.051*	
C6	0.1059 (4)	0.35287 (19)	0.07061 (9)	0.0445 (5)	
H6	0.1171	0.3443	0.0254	0.053*	
C7	−0.4289 (4)	0.61772 (19)	0.15119 (11)	0.0536 (6)	
H7A	−0.3154	0.6726	0.1267	0.080*	
H7B	−0.5402	0.6681	0.1785	0.080*	
H7C	−0.5429	0.5684	0.1213	0.080*	
C8	0.4607 (4)	0.18930 (18)	0.08365 (9)	0.0420 (5)	
H8	0.4667	0.1849	0.0381	0.050*	
C9	0.9333 (4)	−0.05273 (18)	0.11353 (9)	0.0382 (4)	
H2	0.799 (5)	0.058 (2)	0.0411 (5)	0.080*	
H3A	0.998 (4)	−0.1287 (16)	0.1999 (10)	0.080*	
H3B	0.830 (4)	−0.0115 (18)	0.2022 (10)	0.080*	

### Atomic displacement parameters (Å^2^)

**Table:**

	*U*^11^	*U*^22^	*U*^33^	*U*^12^	*U*^13^	*U*^23^
N1	0.0443 (9)	0.0365 (9)	0.0377 (9)	−0.0007 (7)	0.0136 (7)	−0.0049 (7)
N2	0.0523 (10)	0.0419 (9)	0.0331 (9)	0.0090 (7)	0.0157 (8)	−0.0006 (7)
N3	0.0661 (12)	0.0616 (12)	0.0315 (9)	0.0130 (9)	0.0107 (8)	0.0032 (8)
O1	0.0617 (9)	0.0581 (9)	0.0365 (8)	0.0216 (7)	0.0051 (6)	−0.0001 (6)
O2	0.0500 (8)	0.0493 (8)	0.0463 (8)	0.0142 (6)	0.0110 (7)	−0.0011 (6)
S1	0.0681 (4)	0.0523 (3)	0.0400 (3)	0.0193 (3)	0.0167 (3)	0.0030 (2)
C1	0.0411 (10)	0.0370 (10)	0.0336 (10)	−0.0025 (8)	0.0084 (8)	−0.0039 (8)
C2	0.0380 (10)	0.0402 (10)	0.0333 (10)	−0.0004 (8)	0.0049 (8)	−0.0005 (8)
C3	0.0472 (11)	0.0478 (12)	0.0314 (10)	0.0040 (9)	0.0085 (9)	−0.0041 (8)
C4	0.0367 (10)	0.0380 (10)	0.0404 (11)	−0.0010 (8)	0.0085 (8)	−0.0007 (8)
C5	0.0460 (11)	0.0430 (11)	0.0386 (11)	0.0049 (9)	0.0025 (9)	0.0045 (9)
C6	0.0545 (12)	0.0485 (12)	0.0311 (10)	0.0008 (10)	0.0072 (9)	−0.0017 (8)
C7	0.0537 (13)	0.0443 (12)	0.0631 (14)	0.0107 (10)	0.0073 (11)	0.0033 (10)
C8	0.0510 (12)	0.0427 (11)	0.0332 (10)	−0.0005 (9)	0.0114 (9)	−0.0039 (8)
C9	0.0416 (11)	0.0401 (11)	0.0333 (10)	−0.0046 (8)	0.0077 (8)	−0.0021 (8)

### Geometric parameters (Å, °)

**Table:**

N1—C8	1.279 (2)	C1—C2	1.402 (3)
N1—N2	1.382 (2)	C1—C8	1.449 (3)
N2—C9	1.336 (2)	C2—C3	1.374 (3)
N2—H2	0.899 (10)	C3—C4	1.382 (3)
N3—C9	1.321 (2)	C3—H3	0.9300
N3—H3A	0.882 (9)	C4—C5	1.389 (3)
N3—H3B	0.886 (9)	C5—C6	1.381 (3)
O1—C2	1.361 (2)	C5—H5	0.9300
O1—H1	0.8200	C6—H6	0.9300
O2—C4	1.361 (2)	C7—H7A	0.9600
O2—C7	1.432 (2)	C7—H7B	0.9600
S1—C9	1.685 (2)	C7—H7C	0.9600
C1—C6	1.393 (3)	C8—H8	0.9300
			
C8—N1—N2	115.42 (16)	C3—C4—C5	119.83 (17)
C9—N2—N1	121.62 (16)	C6—C5—C4	118.70 (17)
C9—N2—H2	122.0 (15)	C6—C5—H5	120.7
N1—N2—H2	116.4 (15)	C4—C5—H5	120.7
C9—N3—H3A	122.4 (15)	C5—C6—C1	122.82 (18)
C9—N3—H3B	122.7 (15)	C5—C6—H6	118.6
H3A—N3—H3B	114.6 (18)	C1—C6—H6	118.6
C2—O1—H1	109.5	O2—C7—H7A	109.5
C4—O2—C7	118.50 (16)	O2—C7—H7B	109.5
C6—C1—C2	116.84 (17)	H7A—C7—H7B	109.5
C6—C1—C8	119.77 (17)	O2—C7—H7C	109.5
C2—C1—C8	123.39 (17)	H7A—C7—H7C	109.5
O1—C2—C3	116.95 (16)	H7B—C7—H7C	109.5
O1—C2—C1	122.03 (16)	N1—C8—C1	122.81 (18)
C3—C2—C1	121.00 (17)	N1—C8—H8	118.6
C2—C3—C4	120.80 (17)	C1—C8—H8	118.6
C2—C3—H3	119.6	N3—C9—N2	117.57 (17)
C4—C3—H3	119.6	N3—C9—S1	122.11 (15)
O2—C4—C3	115.21 (17)	N2—C9—S1	120.31 (14)
O2—C4—C5	124.97 (17)		

### Hydrogen-bond geometry (Å, °)

**Table:**

*D*—H···*A*	*D*—H	H···*A*	*D*···*A*	*D*—H···*A*
N3—H3B···O2^i^	0.89 (1)	2.26 (2)	2.998 (3)	141.(2)
N3—H3A···O1^ii^	0.88 (1)	2.23 (1)	3.076 (3)	162 (2)
N2—H2···S1^iii^	0.90 (1)	2.48 (1)	3.366 (3)	168 (2)
O1—H1···N1	0.82	1.99	2.700 (2)	145

Symmetry codes: (i) −*x*+1/2, *y*−1/2, −*z*+1/2; (ii) −*x*+3/2, *y*−1/2, −*z*+1/2; (iii) −*x*+2, −*y*, −*z*.
